# Room temperature *in-situ* measurement of the spin voltage of a BiSbTe_3_ thin film

**DOI:** 10.1038/s41598-020-59679-9

**Published:** 2020-02-18

**Authors:** Arthur Leis, Michael Schleenvoigt, Abdur Rehman Jalil, Vasily Cherepanov, Gregor Mussler, Detlev Grützmacher, F. Stefan Tautz, Bert Voigtländer

**Affiliations:** 1 Peter Grünberg Institut (PGI-3), Forschungszentrum Jülich, 52425 Jülich, Germany; 2 Jülich Aachen Research Alliance (JARA), Fundamentals of Future Information Technology, 52425 Jülich, Germany; 3 Experimentalphysik IV A, RWTH Aachen University, Otto-Blumenthal-Straße, 52074 Aachen, Germany; 4 Peter Grünberg Institut (PGI-9), Forschungszentrum Jülich, 52425 Jülich, Germany

**Keywords:** Surfaces, interfaces and thin films, Topological insulators

## Abstract

One of the hallmarks of topological insulators (TIs), the intrinsic spin polarisation in the topologically protected surface states, is investigated at room temperature *in-situ* by means of four-probe scanning tunnelling microscopy (STM) for a BiSbTe_3_ thin film. To achieve the required precision of tip positions for measuring a spin signal, a precise positioning method employing STM scans of the local topography with each individual tip is demonstrated. From the transport measurements, the spin polarisation in the topological surface states (TSS) is estimated as *p* ~ 0.3 – 0.6, which is close to the theoretical limit.

## Introduction

Three-dimensional topological insulators (TI) are known to possess topologically protected surface states (TSS) emerging from time-reversal symmetry and strong spin-orbit coupling in the material^1–3^. Topological surface states are located in the band gap of the TI and form a linearly dispersing Dirac cone. Moreover, these topological surface states are helical in character, so that electrons populating them have a spin that is locked to their momentum^4^. As a consequence of this spin-momentum locking, TI materials in principle allow for the generation of spin-polarised charge carriers merely by the injection of a charge current, without the need of external magnetic fields or ferromagnetic contacts. These and other properties of topological surface states prove to be useful for spintronics^5,6^ and quantum computation^7^. It is therefore important to realize TSS with a large degree of helical polarisation, preferably already at room temperature.

The helical spin texture of the topological surface states of 3D TIs has been studied extensively by means of angle-resolved photoemission spectroscopy (ARPES)^8–12^. Recently, also the electrical detection of the intrinsic spin polarisation in TSS has been reported in transport experiments^13–21^, employing a multi-terminal potentiometric approach with ferromagnetic (FM) contacts. However, most transport investigations suffer from intrinsic difficulties, such as the parasitic contribution to charge transport through electronic states from the bulk and contaminations introduced during the ex-situ fabrication of devices, which can modify the electronic structure of the TI and the TSS and consequently reduce its spin polarisation. One approach to reduce bulk contributions to transport experiments is reducing the sample thickness either by exfoliation or by growing epitaxial thin film systems. There are clear advantages in using epitaxial thin films, as the Fermi level within the band gap of the compound TI can be tuned by alloying different TI systems such as Bi_2_Te_3_ and Sb_2_Te_3_^22–25^. Furthermore, epitaxial growth in UHV in conjunction with four-probe STM offers the possibility to measure transport *in-situ*, i.e. without contaminations.

In this study, we report the *in-situ* electrical observation of the intrinsic spin polarisation of charge carriers in a (Bi_0.06_Sb_0.94_)_2_Te_3_ thin film at room temperature. By using a four-probe STM^26^, we inject charge carriers from non-magnetic (NM) tungsten tips and measure the spin-dependent voltage drop between a FM nickel tip and a NM tip. From this measurement, we determine the intrinsic spin polarisation in the TSS of the TI thin film to be *p* ~ 0.3 – 0.6, which is close to theoretical predictions of the maximum value^27^. The large spin signal in our measurements is attributed to the high quality of the epitaxial films, the *in-situ* measurement with a four-probe STM, excluding any lithographic processes, and the strong suppression of the contribution of trivial bulk states to charge transport due to thin film conditions, which forces the entirety of the induced charge current through the spin-polarised TSS. We enhance the sensitivity of the measurements by determining the relevant probe distances using STM scans.

## Principle of the Spin Voltage Measurement

Using a four-tip STM, four-point resistance measurements on the surface of the TI film are performed *in-situ* by positioning the STM tips on the sample and using them as electrical contacts. All measurements are carried out at room temperature in a UHV environment. Rutherford backscattering spectrometry (RBS) of the TI film composition and a previous investigation of a sample grown in the same system^28^ shows that within the experimental error, the surface Fermi level *E*_F_ of our sample is located at the Dirac point (*E*_F_ = *E*_D_ = 0).

**Figure 1:**
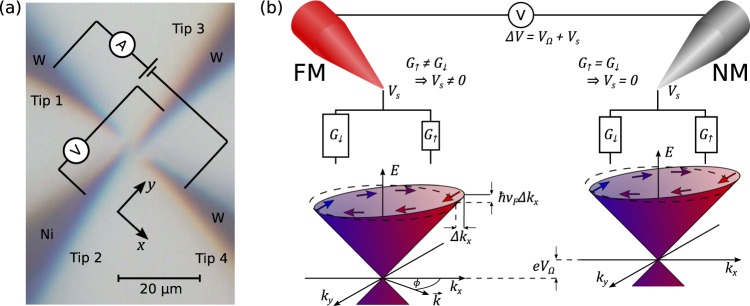
Measurement principle of the spin-dependent potential with STM tips. (**a**) Typical transport measurement setup as seen by the optical microscope. With the four linearly arranged STM tips in contact to the sample surface acting as electrical probes, a current is induced between the outer two tips, while the resulting potential difference is measured between the inner ones. (**b**) Due to the helical nature of the TSS, the orientation of charge carrier spin is perpendicular to their corresponding momentum $\hslash \overrightarrow{k}$ and the surface normal, with the intrinsic TSS spin polarisation *p* being the degree of helicity. The electrical contact between a voltage probe and the TI surface can be regarded as two parallel channels with conductances *G*_↑_ and *G*_↓_ for the two spin orientations. For a finite probe magnetisation (*G*_↑_ ≠ *G*_↓_), the voltage probe acquires a spin-dependent potential *V*_s_, which is given by the interface condition requiring zero current flow (cf. Eq. 1). In this sketch, charge transport takes place in *k*_*x*_-direction and Δ*k*_*x*_ denotes the shift of the Fermi circle due to applied bias. Note that the indicated shift in electrochemical potential $\hslash {v}_{{\rm{F}}}\Delta {k}_{x}\cos \phi$ refers to the extremal position *ϕ* = 0.

**Figure 2:**
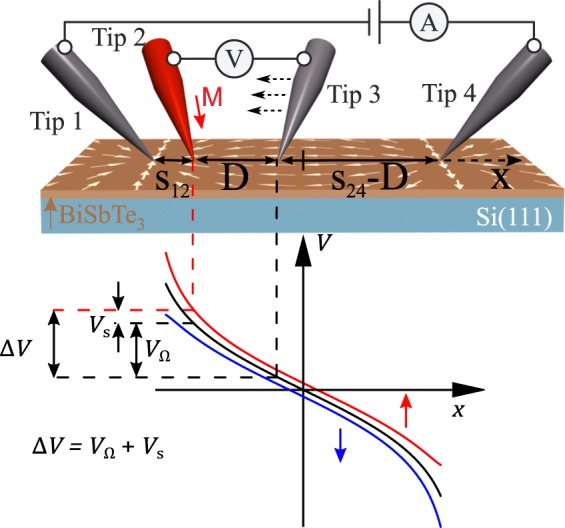
Conceptual sketch of the electrical measurement setup and the resulting potential along the line of the linearly arranged STM tips. While a NM voltage probe senses the local spin-averaged potential indicated by the black line (that is logarithmically-shaped in case of a two-dimensional infinite plane^31^), a magnetised FM tip acquires the spin-chemical potential *V*_s_. As the spin voltage scales with the current density according to Eq. 2, the obtained signal is larger if the FM probe is placed close to a current-injecting tip.

Due to spin-momentum locking, the spin orientation of charge carriers is directly correlated to their momentum. The injection of a current therefore leads to a net spin polarisation of the current carried by the TSS. The STM tips are arranged linearly, such that the distance-dependent measurement of the local electrochemical potential at the surface is enabled, as shown in Figs. 1a and 2. The application of a bias voltage driving the injected current introduces a shift Δ*k*_*x*_ of the Fermi circle in the transport direction from its equilibrium position in reciprocal space. Because of the linear dispersion relation of the TSS, the electrochemical potential of all states at the original Fermi level is shifted by $\hslash {v}_{{\rm{F}}}\Delta {k}_{x}\cos \phi$, where *ϕ* is the angle between the in-plane momentum vector $\overrightarrow{k}$ and the transport direction (Fig. 1b). Due to the spin-momentum locking (indicated by the arrows in Fig. 1b), a shift between the average electrochemical potentials of spin-up and spin-down states is introduced, as long as the intrinsic spin polarisation *p* is finite. Hence, by applying a bias voltage, the current-induced local electrochemical potential is spin-split (Fig. 2).

While a non-magnetic contact can only probe the spin-averaged ohmic potential represented by the black curve in Fig. 2, a FM tip with an effective spin sensitivity *P*_FM_ = (*G*_↑_ − *G*_↓_)/(*G*_↑_ + *G*_↓_)^29^, given by the spin-dependent conductances *G*_↑_ and *G*_↓_ from the FM tip to the sample, can be used to acquire the additional spin-dependent potential component *V*_s_. Figure 1b illustrates the measurement principle. The electrical contact between each voltage probe and the TI surface is represented by two parallel channels for the two spin states. At each point on the TI surface, the voltage probes sit at the potential that is created by the voltage drop between the injection probes. In case of finite *P*_FM_ and finite TSS polarisation *p*, the voltage probe additionally floats to a finite *spin-dependent* potential *V*_s_. For an ideal spin probe *P*_FM_ = 1(−1), *V*_s_ is defined as the average electrochemical potential of charge carriers in the spin-up(-down) state. A general expression for *V*_s_ is obtained from the interface condition requiring zero current flow between the FM voltage probe and the TI surface^30^

1 $${\int }_{0}^{2\pi }{\rm{d}}\phi \left[{G}_{\uparrow }\left\langle \uparrow | \widehat{\rho }| \uparrow \right\rangle +{G}_{\downarrow }\left\langle \downarrow | \widehat{\rho }| \downarrow \right\rangle \right]\left({V}_{{\rm{s}}}-\frac{\hslash {v}_{{\rm{F}}}}{e}\Delta {k}_{x}\cos \phi \right)=0,$$

where the density operator $\widehat{\rho }$ of the statistical ensemble of charge carriers in the Dirac cone depends on their spin polarisation *p*. Hence, $\left\langle \uparrow | \widehat{\rho }| \uparrow \right\rangle$ and $\left\langle \downarrow | \widehat{\rho }| \downarrow \right\rangle$ represent the corresponding probabilities of finding charge carriers in the spin-up and in the spin-down state, respectively. For ideal spin polarisation *p* = 1, the probabilities are given by $\left\langle \uparrow | \widehat{\rho }| \uparrow \right\rangle ={\cos }^{2}\frac{\phi }{2}$ and $\left\langle \downarrow | \widehat{\rho }| \downarrow \right\rangle ={\sin }^{2}\frac{\phi }{2}$. The resulting general expression of the spin potential (as derived in Supplementary Note 4) is given by

2 $${V}_{s}(x)=p{P}_{{\rm{FM}}}\frac{h}{{e}^{2}}j(x){\left[{k}_{{\rm{F}}}+\frac{{k}_{{\rm{B}}}T}{\hslash {v}_{{\rm{F}}}}{\rm{ln}}(1+{e}^{\frac{-{E}_{{\rm{F}}}}{{k}_{{\rm{B}}}T}})\right]}^{-1},$$

with *j*(*x*) representing the local current density at the position of the FM probe. Usually, *V*_s_(*x*) is considered in the limit *E*_F_ ≫ *k*_B_*T*^20,21,30^, resulting in ${V}_{{\rm{s}}}=p{P}_{{\rm{FM}}}\frac{h}{{e}^{2}}\frac{1}{{k}_{{\rm{F}}}}j(x)$, which corresponds to the first term in Eq. 2. In our case, the first term is zero, since *E*_F_ = *E*_D_ = 0 implies *k*_F_ = 0. Therefore, the spin voltage is given by the second term of Eq. 2, as

3 $${V}_{s}(x)=p{P}_{{\rm{FM}}}\frac{h}{{e}^{2}}\left(\frac{\hslash {v}_{{\rm{F}}}}{{k}_{{\rm{B}}}T}\frac{1}{{\rm{ln}}2}\right)j(x),$$

where the term in brackets originates from the effective wave number of thermally excited charge carriers.

An exemplary tip arrangement as seen in optical microscopy is shown in Fig. 1a. Tips 1 and 4 are tungsten STM tips placed at fixed positions to inject a charge current *I* in the TI. Tip 3 is a NM tungsten tip, while tip 2 is a magnetised FM nickel tip. The latter two are used to probe the local electrochemical potential. To magnetise the nickel tip *in-situ*, we use an electromagnet inside the STM chamber. With only one of the two voltage probes being sensitive to the spin orientation of charge carriers, the measured voltage difference between the two comprises an ohmic component *V*_*Ω*_ and a spin-dependent component *V*_s_, as

4 $$\Delta V=\frac{I}{2\pi {\sigma }_{{\rm{2D}}}}\left[{\rm{ln}}\left(\frac{{s}_{24}}{{s}_{12}}\right)-{\rm{ln}}\left(\frac{{s}_{24}-D}{{s}_{12}+D}\right)\right]+{V}_{{\rm{s}}},$$

for purely two-dimensional charge transport^31^. The corresponding distances are depicted in Fig. 2.

According to Eq. 2, the spin voltage *V*_s_ is influenced by material parameters *p* and *v*_F_, the current density $j=\frac{I}{2\pi }\left(\frac{1}{{s}_{12}}+\frac{1}{{s}_{24}}\right)$ at the position of the FM tip^31^ and its *P*_FM_. The spin signal may be obtained in the limit of vanishing inter-tip distance *D* → 0, as in this case the first (ohmic) term *V*_*Ω*_ in Eq. 4 vanishes. In the experiment, we keep tips 1, 2, 4 fixed and move tip 3. By measuring the four-point resistance at various *D* and for opposite FM tip magnetisation directions, we determine the constant spin contribution and estimate the spin polarisation of the surface states of the TI.

## Results

The electrical measurements are performed on the surface of a 10 nm thin film of (Bi_0.06_Sb_0.94_)_2_Te_3_ grown by molecular-beam epitaxy (MBE) on a Si(111) substrate. As the largest spin voltage signal and the highest mobility is expected for a Fermi level close to the Dirac point, we have chosen the corresponding sample stoichiometry. After growth, the sample is transferred to the STM chamber by means of a vacuum transfer. The details of the MBE growth procedure are outlined in a previous publication^32^. The independent measurement of *σ*_2D_ which enters as a parameter in Eq. 4 is presented in Supplementary Note 2.

### Spin voltage measurement

**Figure 3:**
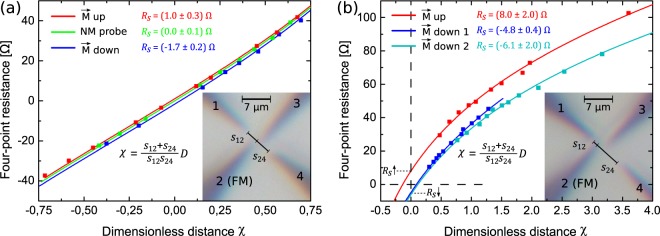
Results of the spin voltage measurement in two tip configurations. The spin-sensitive four-point resistance is measured as function of distance between the inner voltage-probing tips. The tip configuration is depicted in the insets, respectively. For the sake of comparison between the sets of measurements with slightly different *s*_12_ and *s*_24_, the resistance is plotted versus a dimensionless inter-tip distance *χ*. (**a**) Measurement results obtained in the symmetric configuration (*s*_12_ ≈ *s*_24_ ≈ 3.5 *μ*m). Data points in red and blue correspond to measurements with opposite magnetisation directions of the FM tip, while data points coloured in green were obtained with only NM tips, providing a control experiment. (**b**) In the high current density configuration, the FM tip is positioned close to a current-injecting tip (*s*_12_ ≈ 0.4 *μ*m, *s*_24_ ≈ 7 *μ*m). Red and blue/cyan data points denote resistances acquired with reversed magnetic polarisation directions of the FM tip, respectively. The lines of corresponding colour represent fits of the resistance model (Eq. 4).

For each tip configuration, we infer the corresponding four-point resistance from *IV*-characteristics recorded with a maximum induced current of *I* ≈ 50 *μ*A. In order to determine tip distances as precisely as possible, we use a method for tip positioning that employs overlapping STM scans. It is described in more detail below. We use a symmetric configuration with the voltage-probing FM tip positioned approximately in between the current-inducing tips with *s*_12_ ≈ *s*_24_ ≈ 3.5 *μ*m. Figure 3a shows results of resistance measurements corresponding to this tip arrangement. In this figure, the varied inter-tip distance *D* is replaced by a normalised dimensionless distance

5 $$\chi =D\frac{{s}_{12}+{s}_{24}}{{s}_{12}{s}_{24}},$$

because this allows the direct comparison of results obtained for different distances, as explained in Supplementary Note 3. Red and blue data points correspond to sets of measurements for which the FM tips have been magnetised oppositely beforehand. The graph also includes a control measurement with only NM tips represented by green data points. As can be inferred from the fits of the model function in Eq. 4 to the data sets (lines of corresponding colour in Fig. 3a), the resistance curves are offset to each other by

6 $${R}_{{\rm{s}}}=\frac{\Delta V(D\to 0)}{I}=p{P}_{{\rm{FM}}}\frac{h}{2\pi {e}^{2}}\frac{\hslash {v}_{{\rm{F}}}}{{k}_{{\rm{B}}}T}\frac{1}{{\rm{ln}}2}\left(\frac{1}{{s}_{12}}+\frac{1}{{s}_{24}}\right),$$

resulting from *V*_s_ as given by Eq. 3 in the case of a 2D current density. As shown in Fig. 3a, we obtain a finite resistance offset with inverted signs for opposite magnetisation of the FM tip with *R*_s_ = (1.0 ± 0.3) *Ω* and *R*_s_ = (−1.7 ± 0.2) *Ω*. In case of our control experiment with an unpolarised tip, we measure a spin resistance *R*_s_ = (0.0 ± 0.1) *Ω*. When performing the same experiment on a Bi_0.53_Sb_0.47_Te_3_ thin film sample, we do not observe any offset in the data, which is explained by the large *k*_F_ in this sample and the correspondingly small *V*_s_ (Eq. 2). The corresponding data are found in Supplementary Note 5. In general, for TI samples of different materials or doping levels, it is important to keep a low *k*_F_ and a small bulk carrier contribution in order for the spin voltage to be pronounced.

While we observe a reversal of the sign of the resistance offset upon changing the FM’s magnetisation direction, the magnitude of the signal is rather small. The spin-dependent signal can be enhanced using a tip configuration in which the FM probe is situated in a position of high current density, i.e. close (within a few hundred nm) to a current-injecting tip, resulting in a more pronounced spin-splitting (cf. Fig. 2 and Eq. 3). Figure 3b shows three sets of resistance measurements with different FM polarisation. As can be seen, finite resistance offsets with reversed signs are clearly observed at *χ* = 0 for the respective tip magnetisation orientations. With *R*_s_ = (8 ± 2) *Ω* for one magnetisation direction and *R*_s_ = (−4.8 ± 0.4) *Ω* and *R*_s_ = (−6 ± 2) *Ω* for the opposite one, the effect is larger by a factor of  ~ 5 compared to the symmetric configuration in Fig. 3a, as expected from the corresponding increase of the current density.

### Tip positioning with STM images

The determination of the spin signal in our transport measurements is limited by the uncertainty of the four-point resistance arising from the mispositioning of the STM tips on the surface (see Eq. 6). Since our visual control of the tip positions is based on optical microscopy, the position uncertainty is given by the latter’s resolution. Even when assuming an optimistic error of  ±250 nm, the uncertainty of the ohmic part of the resistance arising from tip positioning controlled by the optical microscope would amount to *δR*_*Ω*_ ≈ 8 *Ω* (using Eq. 4), which is about the same magnitude as the spin-dependent signal. Since this uncertainty depends on the local slope of the electrical potential d*R*_*Ω*_/d*x* ∝ *j*(*x*), just as the desired spin signal itself (Eq. 2), this problem cannot be solved by a sophisticated tip arrangement. Therefore, we use a more precise method of tip positioning to measure the spin-split potential.

**Figure 4:**
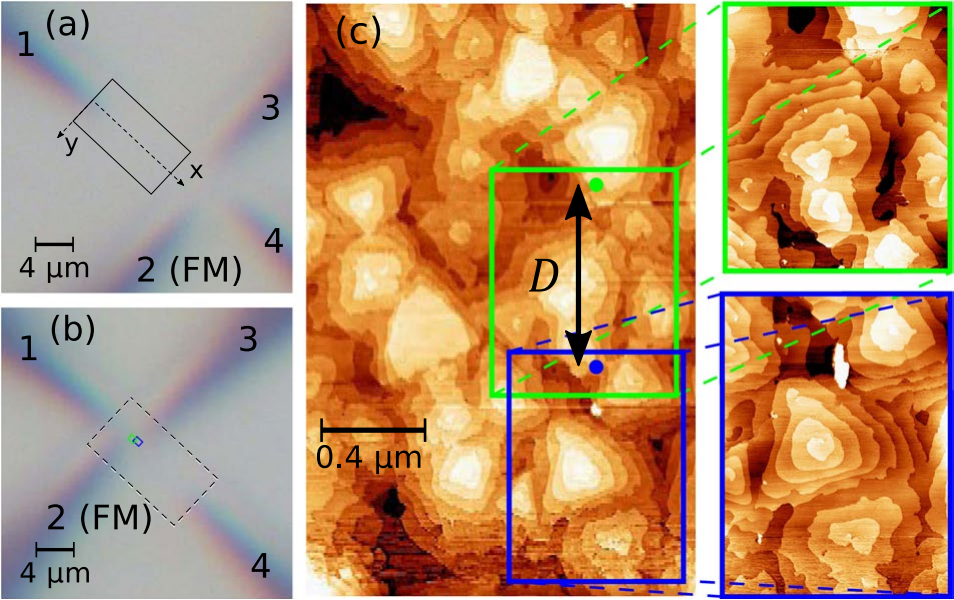
Tip positioning method based on overlapping STM scans used for spin-sensitive transport measurements. (**a**) Using one of the STM tips, a large overview scan of the area of the sample surface in which the contacts will be positioned is acquired. The overview scan (shown partially in (**c**)) constitutes a map for further tip navigation. (**b**,**c**) Subsequently, all four tips are moved close to their target positions to perform small scans. Once a topographic structure from the small scan is recognized in the overview scan, the corresponding tip position within the reference map is known. With all tip positions being identified, the tips can be navigated to their desired configuration in tunnelling contact by using piezoelectric control. The exact position of each tip can then be reconfirmed by further scans before contact is finally established.

Our method of tip positioning employs STM scans of the local sample topography with each of the four tips. In detail, one of the tips (tip 1) is first used to perform an overview scan of a large area (e.g. 4 *μ*m × 10 *μ*m) of the sample surface, while the remaining STM tips reside outside of the scanned area (Fig. 4a). Then, the other tips are moved into the scanned area close to their target positions for the four-probe measurement by use of the optical microscope. Subsequently, all tips are brought into tunnelling contact and small scans of the sample surface are performed, as schematically shown in Fig. 4b. By making use of distinct features in the local surface topography, these scans can be located in the image of the overview scan (Fig. 4c). After moving all tips to their desired positions, the STM tips are lowered to the sample surface to establish electrical contact^31^ and the spin voltage is measured as described above. For a series of distance-dependent four-point measurements, one of the probes is retracted into tunnelling contact and moved to the next position. A possible influence of the step edges seen in the STM scans of the topography on the measured resistance is discussed in Supplementary Note 7.

All inter-tip distances are determined from the tip positions in the large overview scan. As a first approximation, the inter-tips distances can be accessed by measuring the nominal distances between all tip positions in the STM overview scan using the calibrated linear piezo constants of the corresponding piezo elements. However, it is also important to take into account sources of error for the determination of distances in this reference map. Since time-dependent effects such as creep and thermal drift and the non-linear behaviour of the piezoelectric effect at high voltages can distort recorded STM images, we consider the impact of these effects on the overview scan. These effects are evaluated separately, as presented in Supplementary Note 6 and taken into account for the accurate determination of the tip positions. In total, we estimate that the precision for the determination of the tip positions is about  ±25 nm, i.e. at least an order of magnitude better than in optical microscopy. We find that the combined uncertainty from tip positioning results in a systematic error of only few percent for the measured spin resistance *R*_s_.

## Discussion

So far, we obtained the spin-dependent resistance offsets *R*_s_ (Eq. 6) from the four-point measurements. However, these values still depend on the specific tip arrangement, while the spin polarisation of the TSS *p* is the desired intrinsic quantity. Using the recorded STM scans of the TI surface, the inter-tip distances *s*_12_ and *s*_24_ can be determined for each set of measurements. To compare the degree of spin polarisation in our measurements, we define

7 $$S={R}_{{\rm{s}}}/\left(\frac{1}{{s}_{12}}+\frac{1}{{s}_{24}}\right)=p{P}_{{\rm{FM}}}\frac{h}{2\pi {e}^{2}}\frac{\hslash {v}_{{\rm{F}}}}{{k}_{{\rm{B}}}T}\frac{1}{{\rm{ln}}2}$$

as the critical offset parameter, which only depends on intrinsic material parameters. From the measurements in Fig. 3a, we identify *S* = (1.9 ± 0.5) *Ωμ*m and *S* = (−2.9 ± 0.4) *Ωμ*m for the respective FM magnetisation directions, while in the case of the measurements in Fig. 3b, *S* = (3.0 ± 0.8) *Ωμ*m, (−2.9 ± 0.2) *Ωμ*m, (−1.6 ± 0.5) *Ωμ*m are obtained. According to the model introduced above, the *S* stemming from the spin-dependent potential for opposite magnetisations are expected to have the same absolute values but with inverted signs. The observed variation of the absolute values can be explained by the fact that each set of measurements was performed with a different (fresh) FM tip and the effective magnetic tip polarisation *P*_FM_ depends on the microscopic details of each individual tip.

To quantify the spin polarisation *p* in the TSS from our measurements, we make use of the expression for *S* (Eq. 7). We use the Fermi velocity *v*_F_ = 3.8 ⋅ 10^5^ m/s obtained from a previous ARPES measurement of a sample with the same stoichiometry^28^ and take into account a geometrical factor of $1/\sqrt{2}$ in *P*_FM_ due to the 45^°^-orientation of the magnetisation direction, which is along the STM tip, with respect to the surface. Hence, a fully polarised FM tip would yield ${P}_{{\rm{FM}}}=1/\sqrt{2}$ in Eq. 7 in our experimental geometry.

So far, we have only considered the top TSS. Since the current is partially carried by the bottom TSS of the film, only the fraction of the current that propagates through the top TSS is relevant for determining *p*. Assuming that the current divides equally between the top and the bottom TSS and using typical values for the effective spin polarisation of the ferromagnetic tip^33–35^ of *P*_FM_ ~ 0.25–0.5 (without the geometric factor $1/\sqrt{2}$), a spin polarisation in the TSS of *p* ~ 0.3–0.6 results. This range is similar to values observed in other transport investigations^14,16,18,20,36^. The main source of variation of *p* in our experiment is the value of the tip polarisation.

In the literature, the spin polarisation is sometimes defined as the average spin of all charge carriers with a positive group velocity in transport direction^27,37^. Such an averaging over parts of the Fermi circle results in geometric normalisation factors and hence in maximum spin polarisations below 1. For example, in the definition of Yazyev *et al*.^27^, the integration over half the Fermi circle yields a value of *π*/4 for the case of ideal spin texture. In contrast, in our definition of *p* as the intrinsic spin polarisation of the TSS, the maximum value corresponding to an ideal helical spin texture is *p* = 1.

To put our measured value of 0.3–0.6 into perspective, we note that ab initio calculations for TI materials have shown that the TSS polarisation *p* as defined in this work can be reduced by ~0.35 due to spin-orbit entanglement^27^, as the electron spin quantum number is no longer conserved in the corresponding TI systems. Assuming that there is spin-orbit entanglement in our sample, our measured range of *p* is remarkably close to the theoretical limit. Another possible reduction of *p* results from bulk states. Previous investigations have shown that the coexistence of excited bulk states is severely limiting the magnitude of the spin voltage in 3D TIs^14,21,29^. In this regard, our thin film system is beneficial as it has been shown that the bulk contribution to the conductivity is negligible^32,38^ and that the Te interface layer between our TI film and the substrate does not contribute to the measured conductivity^39^.

As suggested in previous publications^30,40^, spin polarisation signals in transport measurements can also be interpreted to have other causes such as the bulk Rashba effect or the local Hall effect induced by the stray field of polarised FM contacts. While the Hall effect can be excluded in our system due to the small dimension of the contact area of our tips and a general absence of the Hall effect in infinite plane systems^41,42^, a contribution of the Rashba effect of bulk states to the spin signal in TI samples is in principle possible^29^. However, the thermal population of the appropriate spin-split bulk states is expected to be small due to a negligible number of bulk charge carriers in our thin film system. Furthermore, any contribution of a Rashba effect caused by charge carriers accumulated at the surface of the TI due to band bending^43^ can be excluded, as the effect of near-surface band bending is negligible in case of a 10 nm film^32,38^. The magnitude of our detected signal also seems to be too large to originate from Rashba-split states, which are expected to contribute less to spin polarisation than the TSS, because of the smaller Fermi circle and a partial suppression from Rashba states with opposite helicity^37^. In general, if there is a bulk Rashba effect concurrent to the TSS, it is expected to be counteracting due to its opposite polarisation^37^.

In conclusion, we presented spin-sensitive, distance-dependent four-point resistance measurements of the spin voltage using a four-tip STM. Our tip positioning technique, relying on overlapping scans with each of the four tips, allows for transport measurements with precisely defined probe geometries. The *in-situ* spin-sensitive resistance measurements on the MBE-grown (Bi_0.06_Sb_0.94_)_2_Te_3_ thin film sample reveal a spin signal at room temperature. With other sources of spin polarisation of charge carriers proven to be irrelevant, we conclude that the effect is caused by spin-momentum locking in the TSS. A quantitative evaluation, assuming an equal distribution of the total current between the top and the bottom TSS, leads to an estimate for the TSS spin polarisation of *p* ~ 0.3–0.6. This range for the spin polarisation is remarkable, as it is close to the theoretical limit for systems with spin-orbit entanglement^27^.

For future applications, BiSbTe_3_ thin films can be grown on gate-controllable substrates^32^, enabling the electrical gate control of the spin voltage by changing the Fermi wave number *k*_F_ of the charge carriers. Furthermore, our tip positioning method offers possibilities to perform transport measurements on structures that require precise electrical contacting, such as the edge channels of 2D TI films.

## Methods

### Sample preparation

The thin-film growth is performed on a 10 × 10 mm^2^ Si(111) sample by means of molecular beam epitaxy (MBE). The Si sample is cleaned from organic contaminations and the native oxide chemically using a wet etching procedure (RCA/hydrofluoric acid). After the transfer to the MBE chamber (base pressure 1 ⋅ 10^−10^ mbar) and prior to deposition, the Si sample is heated to 700 ^°^C for $10\ \min$ to remove the hydrogen termination at the surface that is formed during etching. The subsequent growth of the TI film is carried out at a substrate temperature of 330 ^°^C and evaporator temperatures of *T*_Te_ = 325 ^°^C, *T*_Bi_ = 470 ^°^C and *T*_Sb_ = 475 ^°^C, corresponding to a flux ratio of 100: 10: 1 (Te: Sb: Bi). To ensure the formation of a Te monolayer on the Si(111) surface at the start of the growth process, the Te evaporator is opened a few seconds ahead of the Bi and Sb evaporators. After depositing the thin film and cooling the sample down to room temperature, the sample is loaded into the four-tip scanning tunnelling microscope (STM) chamber by means of vacuum transfer. Subsequent to the *in-situ* measurements, X-ray reflectivity and Rutherford backscattering measurements are performed ex-situ to confirm the film thickness and to determine the atomic composition of the ternary compound.

### Four-probe measurement

The transport investigation is carried out *in-situ* using a four-tip STM at room temperature. Electrical contact to the TI film is established by lowering the four STM tips towards the sample from tunnelling contact. For all measurements, the tips are arranged in a straight line with two tungsten tips injecting the current, while the remaining two tips (tungsten and nickel) measure the voltage drop in between. The distance-dependent four-point resistances are obtained from the resulting *IV*-characteristics at each respective tip position. For macroscopic resistance measurements at large tip distances (~50 *μ*m), the tip positioning is monitored by an optical microscope. In case of resistance measurements at sub-*μ*m distances, the positions of the tips are controlled using overlapping STM scans performed with each tip. All STM tips used in the experiment are prepared by electrochemical etching. The ferromagnetic nickel tip is magnetised *in-situ* in the STM chamber prior to each set of measurements using an electromagnet.

## Supplementary information

Supplementary Information.

## Data Availability

Data within the manuscript and its Supplementary Information is available from the corresponding author upon reasonable request.
