# Supplementary material for: Room temperature in-situ measurement of the spin voltage of a BiSbTe3 thin film
Source: Sci Rep. 2020 Feb 18;10:2816. doi: 10.1038/s41598-020-59679-9 (PMC7029040; doi:10.1038/s41598-020-59679-9)
Supplement: Supplementary file 1 — Supplementary Information. [file 41598_2020_59679_MOESM1_ESM.pdf]

Supplementary Information for

Room temperature in-situ measurement of the spin voltage of a $BiSbTe_3$ thin film

Arthur Leis,[1,2,3] Michael Schleenvoigt,[2,4] Abdur Rehman Jalil,[2,4] Vasily Cherepanov,[1,2] Gregor Mussler,[2,4] Detlev Grützmacher,[2,4] F. Stefan Tautz,[1,2,3] Bert Voigtländer[1,2,3,*]

[1] *Peter Grünberg Institut (PGI-3), Forschungszentrum Jülich, 52425 Jülich, Germany*

[2] *Jülich Aachen Research Alliance (JARA), Fundamentals of Future Information Technology, 52425 Jülich, Germany*

[3] *Experimentalphysik IV A, RWTH Aachen University, Otto-Blumenthal-Straße, 52074 Aachen, Germany*

[4] *Peter Grünberg Institut (PGI-9), Forschungszentrum Jülich, 52425 Jülich, Germany*

Supplementary Note 1: Position of the Fermi level

In order to obtain the position of the Fermi level in the band gap for our thin film, we determine the stoichiometry of the sample and use interpolation of known spectroscopic data of samples grown in the same system.

The exact material composition of the TI film used for the current investigation is determined by means of Rutherford Backscattering Spectrometry (RBS) measurements. From these measurements, we infer a Sb concentration of $x = 0.946 \pm 0.001$. In a prior investigation[1], the position of the Fermi level with respect to the Dirac point was determined to $E_\mathrm{F} - E_\mathrm{D} = (2 \pm 7)\,\mathrm{meV}$ for a $(Bi_{1-x}Sb_x)_2Te_3$ thin film with 0.94 ± 0.01 Sb concentration. In case of a $(Bi_{0.53}Sb_{0.47})_2Te_3$ thin film grown in the same system, the Fermi level is situated at $E_\mathrm{F} - E_\mathrm{D} = 250\,\mathrm{meV}$, i.e. close to the conduction band[2]. Using the fact that the position of the Fermi level with respect to the Dirac point is linearly dependent on the content of Sb in the composite between $\mathrm{x} = 0.5$ and $\mathrm{x} = 1$[3], we estimate a Fermi level shift of $\sim 5\,\mathrm{meV}$ per percent of Sb for varying film stoichiometries in this regime. From this estimation, we find a nominal value of $(-3 \pm 7)\,\mathrm{meV}$ for the position of the Fermi level in our sample, which is assumed as $E_\mathrm{F} \approx 0$ within the experimental accuracy.

We find that the uncertainty $\pm 7\,\mathrm{meV}$ for the position of the Fermi level, according to propagation of uncertainties using Eq. 2, leads to an upward deviation of our resulting estimate

of the intrinsic spin polarisation p of up to ~ 0.09. This deviation is considered insignificant compared to the uncertainty resulting from the value of the tip polarisation.

Supplementary Note 2: TI conductivity

We determine the conductivity of the $(Bi_{0.06}Sb_{0.94})_2Te_3$ thin film by performing four-point resistance measurements at varying probe distances. For this purpose, four NM tungsten tips are positioned in-line by coarse positioning under inspection by the optical microscope, with two of the inter-tip distances kept constant at $s = 50$ µm, while the remaining distance x to one of the outer current-injecting tips is varied in between measurement points, as shown in the inset of Supplementary Fig. 1. The x-dependent four-point resistance obtained from the voltage drop between the inner probes is shown in Supplementary Fig. 1. As is expected for a TI film of 10 nm thickness, the obtained resistance data correspond very well to a two-dimensional conductivity model[5]. The corresponding 2D conductivity is determined as $\sigma_{2D} = 2.86(1)$ mS.

This conductivity is ~ 7 times larger than the conductivity of a $(Bi_{0.53}Sb_{0.47})_2Te_3$ sample measured as $\sigma_{2D} = 0.44(5)$ mS in a previous investigation[2]. At first sight, this seems counter-intuitive since the total TSS charge carrier density of the present sample $(n_{TSS} + p_{TSS}) \approx 2.8 \cdot 10^{11}$ cm^{-2} is smaller than the charge carrier density of the $(Bi_{0.53}Sb_{0.47})_2Te_3$ sample ($n_{TSS} \approx 4 \cdot 10^{12}$ cm^{-2}). For this comparison, the total TSS charge carrier densities for electrons (n_{TSS}) and holes (p_{TSS}) of our TI film are determined by[4]

$$n_{\mathrm{TSS}} = \frac{k_{\mathrm{B}}T}{2\pi}\int_0^\infty dE\,\frac{E}{(\hbar v_{\mathrm{F}})^2}\left(1 + e^{(E-E_{\mathrm{F}})/(k_{\mathrm{B}}T)}\right)^{-1}$$

$$p_{\mathrm{TSS}} = \frac{k_{\mathrm{B}}T}{2\pi}\int_0^\infty dE\,\frac{E}{(\hbar v_{\mathrm{F}})^2}\left(1 + e^{(E+E_{\mathrm{F}})/(k_{\mathrm{B}}T)}\right)^{-1} \quad ,$$

where the position of the Fermi level is $E_F \approx E_D = 0$. In principle, this discrepancy could be explained either by an increased contribution of bulk states to transport or by an increased TSS mobility in our sample. We can rule out the first possibility by the following consideration:

Using the measured difference of 40 meV between the Fermi level and the valence band[1], we can estimate the density of charge carriers populating the bulk states for our finite temperature conditions. Since near-surface band bending has been previously shown to be negligible irrespective of dopant concentration for thin film systems[2,6], we calculate a charge carrier

density of $n_{\mathrm{2D,film}} = 3 \cdot 10^{11}\,\mathrm{cm}^{-2}$ arising from bulk states in the Boltzmann approximation, which corresponds to a small contribution to transport due to the low mobility of $\mu_{\mathrm{film}} < 2\,\mathrm{cm}^2/\mathrm{Vs}$ of bulk states at room temperature[2]. Therefore, we exclude contributions from bulk states and assign the high conductivity obtained from the measurements to an increased TSS mobility of $\mu = \frac{\sigma_{2D}/2}{e(n_{\mathrm{TSS}}+p_{\mathrm{TSS}})} \approx 32{,}000\,\mathrm{cm}^2/\mathrm{Vs}$ under the assumption that the mobility and the position of the Fermi level of the bottom TSS are equal to those of the top TSS. An increased mobility of the TSS is plausible, as previous investigations have also reported a sharp increase of mobility if the Fermi level is located close to the Dirac point[3,7,8].

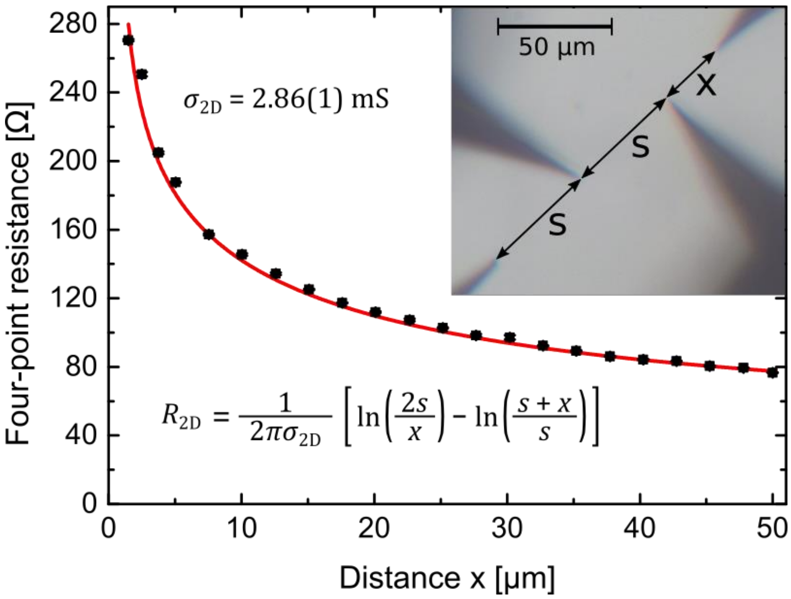

Supplementary Figure 1. Distance-dependent four-point resistance recorded with only NM tungsten tips to identify the conductivity of the investigated TI sample separately from the spin-sensitive transport measurements. The tips were positioned on the surface using the optical microscope. The red line represents a fit of the 2D resistance corresponding to the chosen probe configuration[1] with $s = 50$ µm as a function of inter-tip distance x. The inset depicts an optical microscope image of the respective measurement configuration. As expected for a thin film of 10 nm thickness, the data is fully explained by a two-dimensional conductivity model.

Supplementary Note 3: Dimensionless tip distance

For the presentation of the results of the spin-sensitive resistance measurements, we use a normalised dimensionless inter-tip distance χ to compare the different measurements to each

other. For a given configuration of the fixed tip positions s_{12} and s_{14} between each set of measurements, the distance-dependent four-point resistance is determined by

$$R = \frac{1}{2\pi\sigma_{2D}}\left[\ln\left(\frac{s_{24}}{s_{12}}\right) - \ln\left(\frac{s_{24} - D}{s_{12} + D}\right)\right] + R_s \quad . \qquad \text{(S1)}$$

According to this equation, $\frac{dR}{dD}$ depends on s_{12} and s_{14}. This makes extrapolation of $R(D \rightarrow 0)$ from data points with different s_{12} and s_{14} difficult. Therefore, we use a dimensionless distance χ instead of D.

$$\begin{aligned}\frac{dR}{d\chi} = \frac{dR}{dD}\frac{dD}{d\chi} &= \frac{1}{2\pi\sigma_{2D}}\left(\frac{s_{12} + D}{s_{24} - D}\right)\frac{(s_{12} + D) + (s_{24} - D)}{(s_{12} + D)^2} \cdot \frac{dD}{d\chi} \\ &= \frac{1}{2\,\pi\,\sigma_{2D}}\frac{s_{12} + s_{24}}{(s_{12} + D)(s_{24} - D)} \cdot \frac{dD}{d\chi} \\ &= \frac{1}{2\pi\sigma_{2D}}\frac{s_{12} + s_{24}}{s_{12}s_{24}}\frac{1}{\left(1 + \frac{D}{s_{12}}\right)\left(1 - \frac{D}{s_{24}}\right)} \cdot \frac{dD}{d\chi}\end{aligned}$$

By defining $\chi \equiv \frac{s_{12}+s_{24}}{s_{12}s_{24}} D$, we find $\frac{dD}{d\chi} = \frac{s_{12}s_{24}}{s_{12}+s_{24}}$ and $\frac{dR}{d\chi} = \frac{1}{2\pi\sigma_{2D}}\frac{1}{\left(1+\frac{D}{s_{12}}\right)\left(1-\frac{D}{s_{24}}\right)}$. Since $s_{12}, s_{24} \gg D$, we obtain $\frac{dR}{d\chi} = \frac{1}{2\pi\sigma_{2D}}$, i.e. the slope of the four-point resistance becomes independent of s_{12} and s_{14}.

Supplementary Note 4: Derivation of the spin voltage V_s

The expression for the spin voltage obtained from measuring with a ferromagnetic spin-polarised probe used in the paper is presented here by deriving an equation for the spin voltage taking into account finite temperatures.

As an effect of applied bias, the Fermi circle of the TI Dirac cone is shifted with respect to the equilibrium condition. The relation between the resulting current density and the shift in k-space is determined by

$$\begin{aligned}\vec{j} &= -e\int \frac{d^2k}{(2\pi)^2}\vec{v}(\vec{k})f(\vec{k}) \\ &= -\frac{e}{(2\pi)^2}\int dk_x dk_y\, v_F(\cos\phi\,\vec{e_x} + \sin\phi\,\vec{e_y})\left[f_0(\vec{k}) + \overrightarrow{\Delta k}\cdot\overrightarrow{\nabla_k}f_0(\vec{k})\right] \quad ,\end{aligned}$$

where f_0 is the equilibrium Fermi-Dirac distribution. Assuming $\overrightarrow{\Delta k} \,||\, \overrightarrow{e_x}$ (i.e. $\Delta k = \Delta k_x$) without loss of generality,

$$\vec{j} = -\frac{ev_{\mathrm{F}}}{(2\pi)^2} \int_0^{2\pi} \mathrm{d}\phi \left(\cos\phi\, \overrightarrow{e_x} + \sin\phi\, \overrightarrow{e_y}\right) \int_0^{\infty} \mathrm{d}k\; k\, \Delta k_x \frac{\mathrm{d}f_0}{\mathrm{d}k_x}$$

$$= \frac{ev_{\mathrm{F}}}{(2\pi)^2} \int_0^{2\pi} \mathrm{d}\phi \left(\cos\phi\, \overrightarrow{e_x} + \sin\phi\, \overrightarrow{e_y}\right) \int_0^{\infty} \mathrm{d}k\; k\, \Delta k_x \frac{e^{\frac{\hbar v_{\mathrm{F}} k - E_{\mathrm{F}}}{k_{\mathrm{B}} T}}}{\left(1 + e^{\frac{\hbar v_{\mathrm{F}} k - E_{\mathrm{F}}}{k_{\mathrm{B}} T}}\right)^2} \frac{\hbar v_{\mathrm{F}}}{k_{\mathrm{B}} T} \frac{k_x}{k}$$

$$= \frac{ev_{\mathrm{F}}}{(2\pi)^2} \int_0^{2\pi} \mathrm{d}\phi \left(\cos\phi\; \overrightarrow{e_x} + \sin\phi\, \overrightarrow{e_y}\right) \int_0^{\infty} \mathrm{d}k\; k \cos\phi\;\; \Delta k_x \frac{e^{\frac{\hbar v_{\mathrm{F}} k - E_{\mathrm{F}}}{k_{\mathrm{B}} T}}}{\left(1 + e^{\frac{\hbar v_{\mathrm{F}} k - E_{\mathrm{F}}}{k_{\mathrm{B}} T}}\right)^2} \frac{\hbar v_{\mathrm{F}}}{k_{\mathrm{B}} T} \;\; .$$

Substituting $u = \frac{\hbar v_{\mathrm{F}} k - E_{\mathrm{F}}}{k_{\mathrm{B}} T}$ leads to

$$\vec{j} = \frac{ev_{\mathrm{F}} \Delta k_x}{(2\pi)^2} \int_0^{2\pi} \mathrm{d}\phi \left(\cos^2\phi\, \overrightarrow{e_x} + \sin\phi\, \cos\phi\, \overrightarrow{e_y}\right) \int_{-E_{\mathrm{F}}/k_{\mathrm{B}} T}^{\infty} \mathrm{d}u \left(\frac{E_{\mathrm{F}}}{\hbar v_{\mathrm{F}}} + u \frac{k_{\mathrm{B}} T}{\hbar v_{\mathrm{F}}}\right) \frac{e^{\mathrm{u}}}{(1 + e^{\mathrm{u}})^2}$$

$$= \frac{ev_{\mathrm{F}} \Delta k_x}{4\pi} \overrightarrow{e_x} \left[k_{\mathrm{F}} + \frac{k_{\mathrm{B}} T}{\hbar v_{\mathrm{F}}} \ln\left(1 + e^{\frac{-E_{\mathrm{F}}}{k_{\mathrm{B}} T}}\right)\right] \;\; . \qquad \text{(S2)}$$

With the shift of the Fermi circle in k-space Δk_x, the occupied states of the TI Dirac cone are tilted according to the dispersion relation. To relate this shift in k-space to the spin potential with respect to the Fermi level V_{s} measured by a ferromagnetic probe with sensitivity P_{FM}, one needs to consider the interface condition at the FM contact, which requires that there is no net current flow between the FM probe and the TI surface[9]

$$\int_0^{2\pi} \mathrm{d}\phi \left(\mathrm{G}_\uparrow |\langle \uparrow |\Psi\rangle|^2 + \mathrm{G}_\downarrow |\langle \downarrow |\Psi\rangle|^2\right) \left(V_{\mathrm{s}} - \frac{\hbar v_{\mathrm{F}}}{e} \Delta k_x \cos\phi\right) = 0 \quad , \qquad (S3)$$

where the spin-dependent conductance values $\mathrm{G}_\uparrow$ and $G_\downarrow$ are multiplied by the probability to find a charge carrier in the respective spin state. The expression in the second pair of brackets is the local difference between the spin potential acquired by the FM probe and the tilted Fermi circle in k-space. The spin wave function $|\Psi\rangle$ of the charge carriers in the Dirac cone of the TI is determined from spin-momentum locking and is given by $|\Psi\rangle = \cos\frac{\phi}{2} |\uparrow\rangle + \sin\frac{\phi}{2} |\downarrow\rangle$ for the case of 100% spin polarisation. In case of zero spin polarisation, the probability to find a

charge carrier in the spin up or down state does not depend on ϕ and is $\frac{1}{2}$ all around the Dirac cone.

For a polarisation $0 \leq p < 1$, the spin state of charge carriers cannot be written as a pure state and therefore, a statistical ensemble of spin states at fixed $\vec{k}$ described by a density matrix $\hat{\rho} = P_\Psi|\Psi\rangle\langle\Psi| + P_{\widetilde{\Psi}}|\widetilde{\Psi}\rangle\langle\widetilde{\Psi}|$ must be considered, where $|\widetilde{\Psi}\rangle = -\sin\frac{\phi}{2}\,|\uparrow\rangle + \cos\frac{\phi}{2}\,|\downarrow\rangle$ is the orthogonal state to the spin state $|\Psi\rangle$ (such that $\langle\widetilde{\Psi}|\Psi\rangle = 0$). The probabilities $P_\Psi = \frac{1+p}{2}$ and $P_{\widetilde{\Psi}} = \frac{1-p}{2}$ of finding a charge carrier in the respective spin state are linked to the spin polarisation of the TSS such that $p = 1$ results in all of the charge carriers in the ensemble being in the pure spin state corresponding to the TI helicity, while $p = 0$ yields a fully mixed incoherent superposition with its orthogonal state ($p = -1$ corresponds to finding all charge carriers in the orthogonal state $|\widetilde{\Psi}\rangle$).

The general expression for the density operator $\hat{\rho}$ of the mixed spin state equates to

$$\hat{\rho} = \begin{pmatrix} \frac{1}{2} - \frac{p}{2}\left(\sin^2\frac{\phi}{2} - \cos^2\frac{\phi}{2}\right) & \frac{p}{2}\sin\phi \\ \frac{p}{2}\sin\phi & \frac{1}{2} + \frac{p}{2}\left(\sin^2\frac{\phi}{2} - \cos^2\frac{\phi}{2}\right) \end{pmatrix}$$

written in the basis of $|\uparrow\rangle$ and $|\downarrow\rangle$. After determining the probabilities $\langle\uparrow|\hat{\rho}|\uparrow\rangle$ and $\langle\downarrow|\hat{\rho}|\downarrow\rangle$ of finding a charge carrier of the ensemble in the spin up and the spin down state, using Eq. S3 yields

$$\int_0^{2\pi} \mathrm{d}\phi\, [\mathrm{G}_\uparrow\langle\uparrow|\hat{\rho}|\uparrow\rangle + \mathrm{G}_\downarrow\langle\downarrow|\hat{\rho}|\downarrow\rangle]\left(V_\mathrm{s} - \frac{\hbar v_\mathrm{F}}{e}\Delta k_x \cos\phi\right)$$

$$= \int_0^{2\pi} \mathrm{d}\phi \left[\mathrm{G}_\uparrow\left(\frac{1}{2} - \frac{p}{2}\left(\sin^2\frac{\phi}{2} - \cos^2\frac{\phi}{2}\right)\right) + \mathrm{G}_\downarrow\left(\frac{1}{2} + \frac{p}{2}\left(\sin^2\frac{\phi}{2} - \cos^2\frac{\phi}{2}\right)\right)\right]\Big(V_\mathrm{s} - \frac{\hbar v_\mathrm{F}}{e}\Delta k_x \cos\phi\Big)$$

$$= V_s\pi(\mathrm{G}_\uparrow + \mathrm{G}_\downarrow) - \frac{\hbar v_\mathrm{F}}{e}\Delta k_x \int_0^{2\pi} \mathrm{d}\phi\, \left[\mathrm{G}_\uparrow\left(-\frac{p}{2}\right) + \mathrm{G}_\downarrow\frac{p}{2}\right]\left(\sin^2\frac{\phi}{2} - \cos^2\frac{\phi}{2}\right)\cos\phi$$

$$= V_s\pi(\mathrm{G}_\uparrow + \mathrm{G}_\downarrow) - \frac{\hbar v_\mathrm{F}}{e}\frac{p}{2}\Delta k_x\pi(\mathrm{G}_\uparrow - \mathrm{G}_\downarrow) \overset{!}{=} 0 \quad .$$

This condition results in the identification of the spin potential

$$V_\mathrm{s} = pP_\mathrm{FM}\frac{\hbar v_\mathrm{F}}{2e}\Delta k_x \quad ,$$

where $P_\mathrm{FM} = (\mathrm{G}_\uparrow - \mathrm{G}_\downarrow)/(\mathrm{G}_\uparrow + \mathrm{G}_\downarrow)$ denotes the effective magnetisation of the FM voltage probe. Using Eq. S2 leads to

$$V_\mathrm{s} = pP_\mathrm{FM}\frac{h}{e^2}j\left[k_\mathrm{F} + \frac{k_\mathrm{B}T}{\hbar v_\mathrm{F}}\ln\left(1 + e^{\frac{-E_\mathrm{F}}{k_\mathrm{B}T}}\right)\right]^{-1} \quad . \quad \text{(S4)}$$

In the limit $E_\mathrm{F} \gg k_\mathrm{B}T$, this general expression reduces to $V_\mathrm{s} = pP_\mathrm{FM}\frac{h}{e^2}\frac{1}{k_\mathrm{F}}j$, which is the form usually given in spin voltage investigations[8,10,11]. For our sample system, the Fermi level is located at the Dirac point within the uncertainty margin of 7 meV. Considering our room temperature conditions, we can assume $E_\mathrm{F} \ll k_\mathrm{B}T$. Therefore, in our case Eq. S4 reduces to

$$V_\mathrm{s} = pP_\mathrm{FM}\frac{h}{e^2}\frac{\hbar v_\mathrm{F}}{k_\mathrm{B}T}\frac{1}{\ln 2}\, j.$$

Supplementary Note 5: Spin-sensitive measurements on $(Bi_{0.53}Sb_{0.47})_2Te_3$

We also performed the spin-sensitive four-point resistance measurements on a 10 nm film with composition $(Bi_{0.53}Sb_{0.47})_2Te_3$. We positioned the STM tips in a symmetric configuration $s_{12} \approx s_{24}$ (cf. inset Supplementary Fig. 2), with the distance between the current-injecting tips being 28 µm and 38 µm for the two recorded sets of measurements, respectively. The results are shown in Supplementary Fig. 2. Contrary to the investigation of the TI thin film presented in the paper, we do not obtain a finite resistance offset for $\chi \rightarrow 0$ for both magnetisation directions, as is evident from the fits of Eq. S1. Considering the material parameters of the thin film, this result is not surprising, since the Fermi wave number $k_\mathrm{F} \approx 0.07$ Å^{-1} of $(Bi_{0.53}Sb_{0.47})_2Te_3$ as determined from ARPES measurements[2] results in a spin signal which is approximately 10 times smaller than that of the TI film investigated in the paper according to Eq. 2. Consequently,

the expected resistance offset resulting from the spin voltage of a perfectly polarised TSS would be $R_s \approx 0.2\,\Omega$, which is smaller than the uncertainty of our measurements.

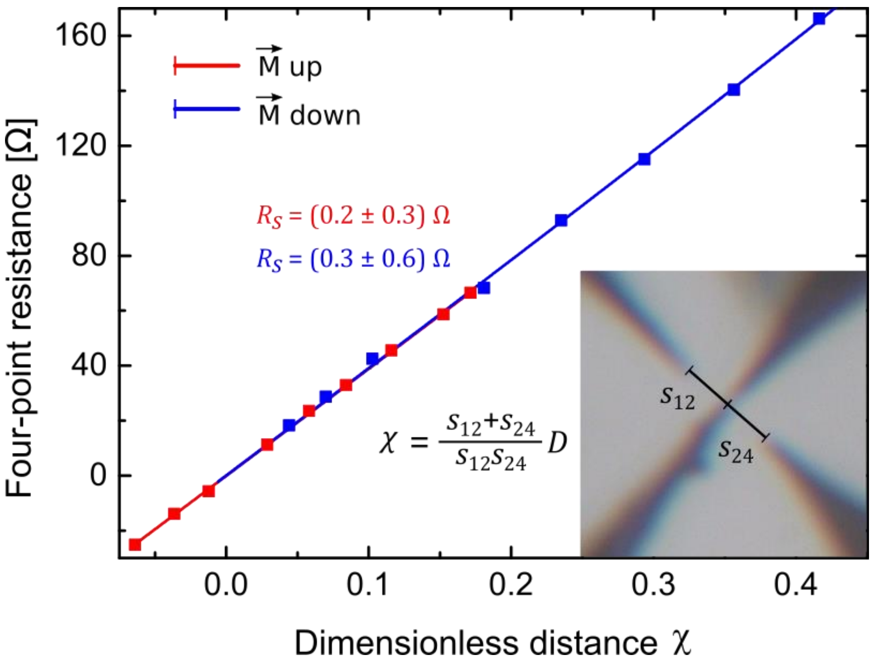

Supplementary Figure 2. Results of the spin voltage measurement of a $(Bi_{0.53}Sb_{0.47})_2Te_3$ thin film. The spin-sensitive four-point resistance is measured as function of the dimensionless distance χ between the inner voltage-probing tips. Data points in red and blue correspond to measurements with opposite magnetisation directions of the FM tip. The lines of corresponding colour represent fits of the resistance model from Eq. S1.

Supplementary Note 6: Details of the tip positioning method

For our investigation of the spin voltage in the TI sample we arrange our STM tips in a desired configuration on the surface by using STM scans of the local topography to identify the position at each tip. As described in the paper, a large overview STM scan serves as a common reference map to determine inter-tip distances. In the following, we consider position accuracies of the apparent positions in the overview scan. Position accuracies in the smaller scans are not relevant, since the determination of tip positions is made via the identification of topographic features in the overview scan.

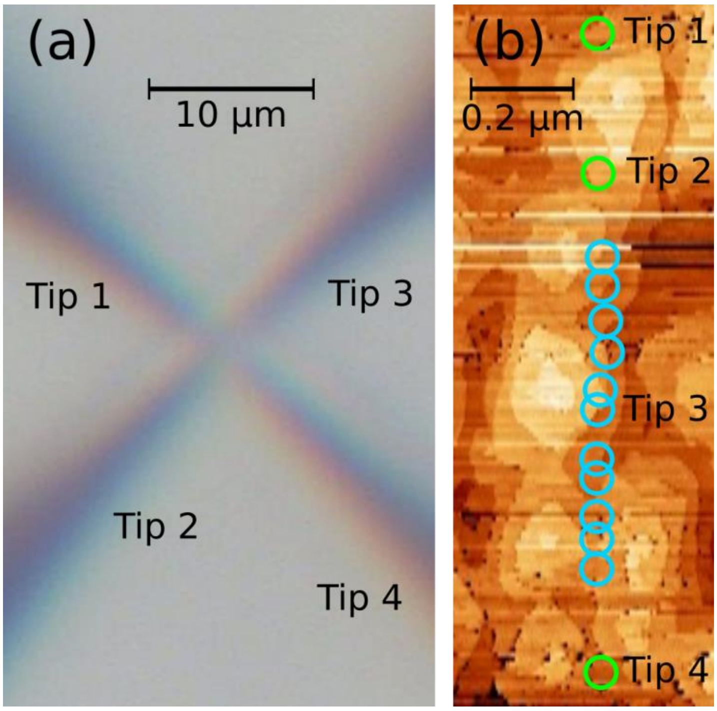

Supplementary Figure 3. Exemplary tip arrangement achieved by positioning via STM scans. (a) Optical microscope image of a four-probe configuration where all tips are arranged in-line within just 1.2 µm. The exact positions of the tips are well below the resolution limit of the optical microscope. **(b)** Corresponding view of the setup in the STM overview scan with the tip positions being marked after identifying them by means of an overlapping scan performed with each respective tip. Cyan circles denote measurement positions of the NM voltage probe which is moved in a series of distance-dependent four-probe measurements. The distances between the marked positions are read off from the as measured overview scan, rescaled using the calibration of the piezoelectric element and used as the inter-tip distances s_{12}, s_{24} and D.

Using this positioning technique, we are able to achieve probe configurations that are impossible to realize with optical control, as shown exemplarily in Supplementary Fig. 3, where all four tips are arranged within just 1.2 µm with additional room for distance variation between the inner tips. The feasible probe distances are limited only by the respective tip radii.

When using the reference map to determine inter-tip distances, it is essential to consider sources of error. Creep, thermal drift, the non-linear behaviour of the piezoelectric element at high voltages and hysteresis are effects that can distort the overview scan. To estimate the impact on our determination of tip distances, we evaluate these effects in the following.

Creep is an effect that only persists for a relatively short time after applying a voltage to the scanning piezo. Therefore, we can exclude any impact on our distance measurement, since all tips are positioned at the centre of the overview scan while creep effects only affect the top of the overview image (fast scan direction: left to right, slow scan direction: top to bottom). Also, creep is minimized by waiting for a sufficient amount of time before starting the overview scan. The effect of thermal drift in our experiment is observed to be constant at < 0.3 nm/min in the

xy-plane for the relevant time scale of the overview scan[5]. Compared to the vertical scanning speed of $\sim 17\,\mathrm{nm/min}$, the relative uncertainty of inter-tip distances arising from this effect amounts to less than 2%. Therefore, thermal drift as a cause of scan distortion is regarded as insignificant.

The non-linearity in the response of the piezoelectric element results in a displacement of the tip and from the nominal position and causes hysteresis at high voltages. When a defined voltage is applied at the scanning piezo (using a nominal displacement of the tip in the xy-plane as defined by the low voltage piezo constant calibrated from images with atomic resolution), the actual displacement of the tip may differ as the piezoelectric response depends on the applied voltage. Since the range of xy-displacement of the STM tip is large in case of the overview scans that we use to determine inter-tip distances, we expect piezo non-linearity to have an influence. Indeed, one can see a locally varying degree of distortion in the large scan images. While we can arrange our four STM tips in-line along the x-dimension for the resistance measurements to make distortion along the y-direction insignificant, it is essential to quantify the effect of distortion arising from piezoelectric non-linearity in order to reconstruct correct distances measured from the overview scan.

Hence, we emulate the x-motion of the tip during the overview scan by applying a voltage to the scanning piezo from the same starting voltage and with the same speed (V/s) as it is applied during a scan and take several images with the optical microscope observing the tip during this motion. These images are used to evaluate the relation between the applied voltage and the distance moved. For this purpose, the relative tip position in each microscope image is determined by fitting an empiric model function to a line profile which is defined along the x-dimension of the tip displacement, as illustrated in Supplementary Fig. 4 (a) and (b). With this approach, we make use of the fact that while the optical microscope is not useful to identify absolute positions of one tip, relative changes in the tip's positions to each other in the images can be determined more precisely. By evaluating the tip's position relative to the starting point over the x-dimension of the entire overview scan range, we obtain the relation between nominal displacement of the scanning piezo and actual displacement of the tip, which exhibits the characteristic non-linear behaviour of piezoelectric elements as seen in Supplementary Fig. 4 (c). Since the overview scan is initiated according to the same protocol prior to every set of resistance measurements, we can calibrate the x-axis of the entire overview scan according to the non-linear model function obtained from this characterization of the scanning piezo. The

reconstructed x-axis of the scan image can then be used to read off the various inter-tip distances of the four-point resistance measurements.

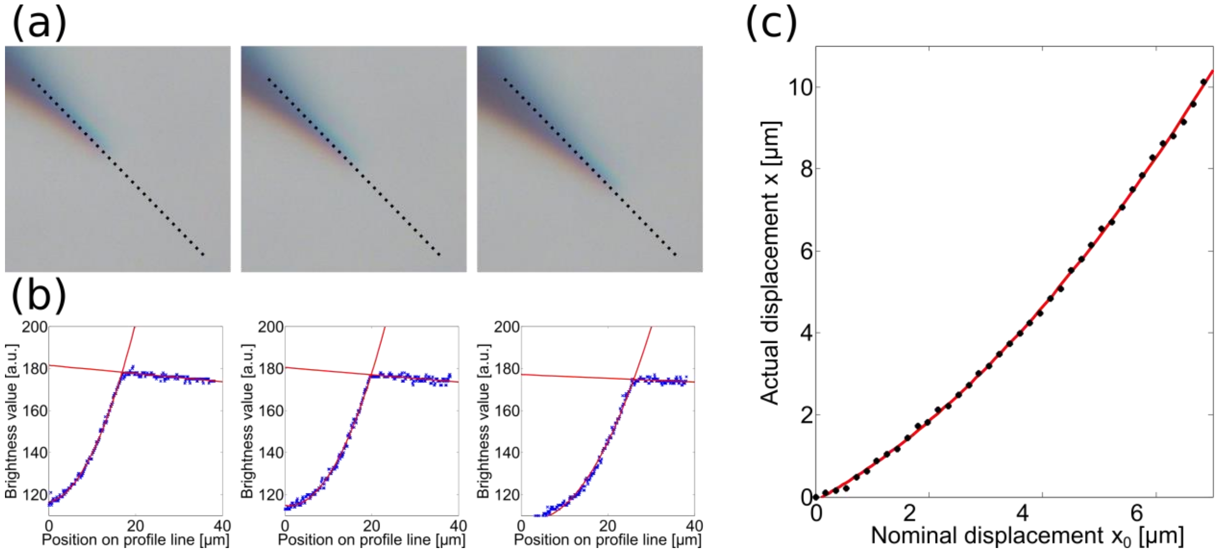

Supplementary Figure 4. Calibration procedure of the piezoelectric element used for the overview scan. (a) The x-movement of the tip during an overview scan is emulated by applying a voltage corresponding to the scanning speed to the piezoelectric element. At various stages of tip displacement, optical microscope images are taken for further image analysis. **(b)** For each microscope image taken, the actual tip position relative to the starting point of the overview scan protocol is determined quantitatively along the dimension of x-displacement by fitting a model function for the cross-sectional tip shape to the brightness values of the image. **(c)** A plot of the quantitatively obtained displacement x vs. the nominal displacement x_0 of the tip reveals the non-linear nature of the piezoelectric response, which can be modeled using a polynomial function. The nominal tip displacement is inferred from the voltage applied to the scanning piezo by multiplication with the (low voltage) piezo constant. With this method the relative tip displacements can be determined very precisely from optical microscope images.

For the calibration of the scanning piezo in this way, we use a second order polynomial function $x = ax_0^2 + bx_0 + c$ to describe the relation between actual tip displacement x and nominal tip displacement x_0, where both x and x_0 are measured from the starting position of the overview scan. The resulting uncertainty of the parameters from the fit of the model function may have a significant influence on the determination of tip distances through the propagation of uncertainties.

The propagation of uncertainties results in a systematic error in tip distances, since e.g. a parameter that is too small would result in a systematic underestimation of all determined tip distances. We quantify this effect for our measurements by additionally determining all tip distances from the polynomial model function with its parameters shifted to the boundaries of the uncertainty margin from the fit value ($a \pm \sigma_a, b \pm \sigma_b, c \pm \sigma_c$). This results in all data points being shifted systematically towards smaller or larger tip distances, respectively. The effect is

greater for larger tip distances. Fits of Eq. S1 to the shifted data are shown in Supplementary Fig. 5 represented by the solid lines of corresponding colour. For comparison, the original data points and the corresponding fit results are included as dots and dashed lines, respectively.

From the fit results, we find that the parameter S defined in the paper changes by only a few percent due to the shift of all inter-tip distances. Therefore, the influence of the systematic error originating from the piezo calibration is regarded as small.

The uncertainty of tip positions $\sigma_x = \sqrt{x_0^4\sigma_a^2 + x_0^2\sigma_b^2 + \sigma_c^2}$ resulting from non-linear behaviour of the piezo element depends on the nominal position x_0 in the overview scan. For the range of nominal tip positions relevant for our study, we extract a mean positioning accuracy of $\sigma_x \approx 25$ nm.

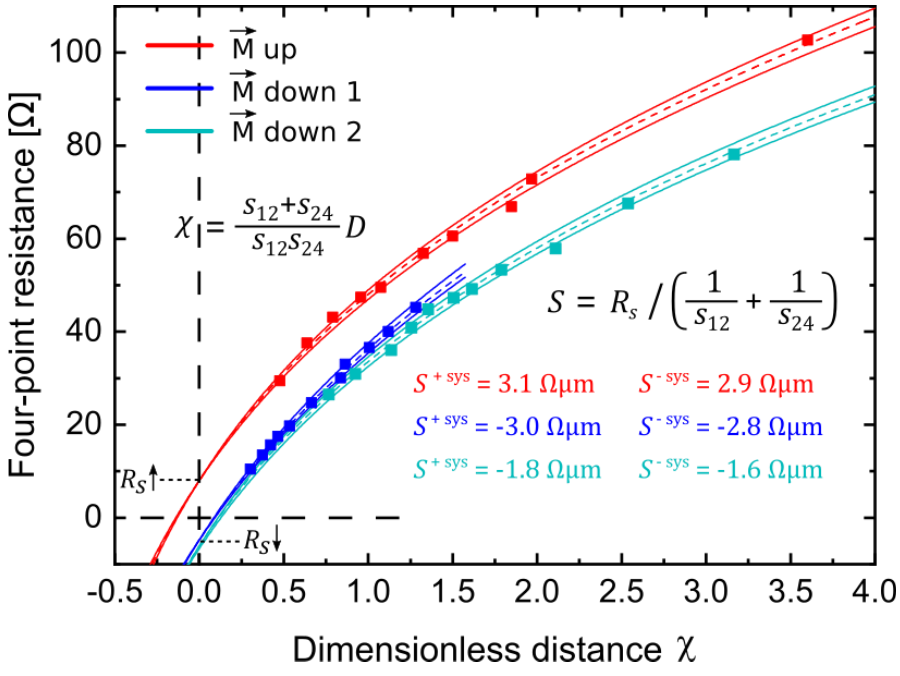

Supplementary Figure 5. Evaluation of the effect of piezo calibration uncertainty on the spin-sensitive measurement results. The uncertainty arising from the determination of the non-linearity of the piezo element causes a systematic shift of all determined tip distances to smaller/larger distances. The magnitude of the resulting systematic error of the spin-polarisation is determined by fitting Eq. S1 to the systematically shifted data (solid lines). The dashed lines represent the fits corresponding to the original data. Since only the parabolic parameter a has an influence on the fit result and the distances between the voltage-probing tips are concentrated to a small range of $\sim 1\,\mu$m (therefore being largely unaffected by the parabolic component of the piezo movement), the systematic error of the spin polarisation turns out to be small.

Supplementary Note 7: Influence of step conductivity on the spin voltage

When looking at the topography of the investigated topological insulator film in the STM scans (e.g. Supplementary Fig. 3 (b)), it can be seen that the surface is not flat, as it consists of terraces at different height levels with step edges in between. One interesting question is whether individual step edges have an effect on the measured four-point resistance. If the corresponding resistance across two terraces has a significant contribution to the total resistance of the film, discrete jumps in the distance-dependent resistance would be expected when traversing step edges with the voltage probing tip. In our data, such systematic changes of resistance cannot be seen.

In a previous investigation of a $(Bi_{0.53}Sb_{0.47})_2Te_3$ thin film grown in the same MBE system, step edges have been found to have a measurable contribution to the electrical resistance[12]. In the nanoscale transport measurements obtained by scanning tunnelling potentiometry, the influence of individual steps can be seen by a voltage drop ΔV_{step} directly across the step edges. The corresponding step conductivity is determined as $\sigma_{\mathrm{step}} = 907\,\mathrm{Scm}^{-1}$. Assuming the same conductivity value for individual steps in our $(Bi_{0.06}Sb_{0.94})_2Te_3$ film, we can estimate the resulting resistance across a step edge for our transport measurements according to

$$\begin{aligned} R_{\mathrm{step}} &= \frac{\Delta V_{\mathrm{step}}}{I} \\ &= \frac{j(x)}{\sigma_{\mathrm{step}}} \cdot \frac{1}{I} \\ &= \frac{1}{2\pi} \frac{d}{(d/2 - x)(d/2 + x)} \cdot \frac{1}{\sigma_{\mathrm{step}}} \quad . \end{aligned}$$

In this expression, d is the distance between the current-injecting tips and x is the position of the step edge along the corresponding connection line measured from centre between the injection points. In our measurements, with $d \approx 7$ µm and $x \approx 2.5$ µm, the expected resistance across a step edge is $R_{\mathrm{step}} \approx 2\,\Omega$. The fact that this value is within the error of a single data point from the model resistance function explains why there is no significant contribution of step conductivities seen in the four-point measurements.

Supplementary References

1. Kellner, J. et al. Tuning the Dirac point to the Fermi level in the ternary topological insulator $(Bi_{1-x}Sb_x)_2Te_3$ *Appl. Phys. Lett.* **107**, 251603 (2015)
2. Lüpke, F. et al. In situ disentangling surface state transport channels of a topological insulator thin film by gating. *npj Quantum Materials* **3**, 46 (2018).
3. Zhang, J. et al. Band structure engineering in $(Bi_{1-x}Sb_x)_2Te_3$ ternary topological insulators. *Nat. Comm.* **2**, 574 (2011)
4. Fang, T., Konar, A., Xing, H., Jena, D. Carrier statistics and quantum capacitance of graphene sheets and ribbons. *Appl. Phys. Lett.* **91**, 092109 (2007)
5. Voigtländer, B., Cherepanov, V., Korte, S., Leis, A., Cuma, D., Just, S. and Lüpke, F. Invited Review Article: Multi-tip Scanning Tunneling Microscopy: Experimental Techniques and Data Analysis. *Rev. Sci. Instruments* **89**, 101101 (2018).
6. Just, S., Lüpke, F., Korte, S., Cherepanov, V., Tautz, F. S. and Voigtländer, B. Parallel conduction channels in topological insulator thin films: Role of the interface layer and the band bending in the film. arXiv:1908.09412v1 [cond-mat.mes-hall] (2019)
7. Kim, D., Cho, S., Butch, N., Syers, P., Kirshenbaum, K., Adam, S., Paglione, J. and Fuhrer, M. Surface conduction of topological Dirac electrons in bulk insulating Bi_2Se_3. *Nat. Phys.* **8**, 459-463 (2011)
8. Ko, W., Nguyen, G. D., Kim, J. S., Zhang, X.-G. and Li, A.-P. Accessing the Intrinsic Spin Transport in a Topological Insulator by Controlling the Crossover of Bulk-to-Surface Conductance. *Phys. Rev. Lett.* **121**, 176801 (2018)
9. Li, P. and Appelbaum, I. Interpreting current-induced spin polarization in topological insulator surface states. *Phys. Rev. B* **93**, 220404 (2016).
10. Hus, S. M., Zhang, X.-G., Nguyen, G. D., Ko, W., Baddorf, A. P., Chen, Y. P. and Li, A.-P. Detection of the Spin-Chemical Potential in Topological Insulators Using Spin-Polarized Four-Probe STM. *Phys. Rev. Lett.* **119**, 137202 (2017)
11. Hong, S., Diep, V., Datta, S. and Chen, Y. P. Modeling potentiometric measurements in topological insulators including parallel channels. *Phys. Rev. B* **86**, 085131 (2012)
12. Lüpke, F. et al. Electrical resistance of individual defects at a topological insulator surface. *Nat. Comm.* **8**, 15704 (2017).
